# Optimizing Concentration of Polyethelene Glycol for Exosome Isolation from Plasma for Downstream Application

**DOI:** 10.3390/medicina58111600

**Published:** 2022-11-04

**Authors:** Marut Tangwattanachuleeporn, Phijitra Muanwien, Yothin Teethaisong, Poorichya Somparn

**Affiliations:** 1 Faculty of Allied Health Sciences, Burapha University, Chon Buri 20130, Thailand; 2 Research Unit for Sensor Innovation, Burapha University, Chon Buri 20130, Thailand; 3 Center of Excellence in Systems Biology, Faculty of Medicine, Chulalongkorn University, Bangkok 10330, Thailand; 4 Center of Excellence on Translational Research in Inflammation and Immunology (CETRII), Department of Microbiology, Faculty of Medicine, Chulalongkorn University, Bangkok 10330, Thailand

**Keywords:** serum, exosome, polyethelene glycol, miRNA, cell free DNA, proteomics

## Abstract

Background: Exosomes are ubiquitous extracellular nanovesicles secreted from almost all living cells that are thought to be involved in several important cellular processes, including cell–cell communication and signaling. Exosomes serve as a liquid biopsy tool for clinical and translational research. Although many techniques have been used to isolate exosomes, including ultracentrigation, size-exclusion chromatography, and immunocapturing-based techniques, these techniques are not convenient, they require expensive instrumentation, and they are unhandy for clinical samples. Precipitation techniques from available commercial kits that contain polyethelene glycol (PEG) are now widely used, but these kits are expensive, especially if a large number of biological samples are to be processed. Objective: the purpose of this study is to compare and optimize the efficacy of different concentrations of PEG with two commercial kits ExoQuick (SBI) and Total Exosome Isolation (TEI) from Invitrogen in human plasma. Methods and Materials: we determined exosome quantity, size distribution, marker expression, and downstream application. Results: among the precipitation methods, we found the size of particles and concentrations with 10–20% PEG are similar to ExoQuick and better than TEI. Interestingly, we detected cfDNA with ExoQuick and 10–20% PEG but not TEI and 5% PEG. Moreover, 10% PEG detection of miR-122 and miR-16 expression was superior to ExoQuick and TEI. Furthermore, in proteomics results it also found the identified proteins better than commercial kits but there was a high level of contamination of other proteins in serum. Conclusions: together, these findings show that an optimal concentration of 10% PEG serves as a guide for use with clinical samples in exosome isolation for downstream applications.

## 1. Introduction

Extracellular vesicles (EVs) are heterogeneous, membrane-bound vesicles. They can be classified based on morphological features into three groups: apoptotic bodies, microvesicles, and exosomes [1]. Exosomes are nano-sized vesicles approximately 50–150 nm in diameter that contain transcription factors, enzymes, extracellular matrix proteins, lipids, and nucleic acids (DNA, mRNA, and miRNA), and they are secreted by several living cells to play important roles in cell–cell communication, as well as in both physiological and pathological processes [1]. Due to exosomes carrying numerous vital active substances, for instance, miRNA, cell free DNA, and proteins, they are among the valuable sources of potential biomarkers for detection of diseases such as cancer and autoimmune diseases.

Ultra-centrifugation (UC) is one of the most commonly used techniques for isolation of exosomes, but this technique is time-consuming, has a lack of reproducibility, and requires expensive equipment. Therefore, this UC method is not suitable for clinical application. Other techniques that rely on ultrafiltration and size exclusion chromatography can impair the membrane integrity of the exosome, resulting in leaking of internal components of the exosome and potentially affecting downstream analysis [2]. Finally, precipitation solution is a proper method for clinical samples because it is an easy and fast approach for exosome isolation which is mostly carried out with commercial kits, but these kits are expensive. In these commercial kits, the reagents contain a polymer such as polyethelene glycol (PEG). PEG has been employed for concentration and purification of viruses for years [3]. Exosomes and virus particles are similar in their biophysical properties. Therefore, a PEG precipitation method can be adopted to isolate, concentrate, and purify exosomes for an inexpensive and efficient alternative approach to commercially available products. Further, PEG could also be a method of choice for preparations of a small and limited quantity of samples, such as plasma and serum, rather than large sample volumes. 

To address this issue, we isolated EVs from pool plasma samples of healthy donors using precipitation methods (final concentrations of PEG/ 1 M NaCl, ExoQuick, and TEI) and compared the exosome yield and size distribution, protein marker presence, cfDAN, miRNA, and protein contents. We carried out the characterization of miRNA and cfDNA by qPCR assays and of protein by mass spectrometry. Here we present a method of unbiased and reproducible strategies for exosome isolation, analyzing the cfDNA, miRNA, and protein contents, which is suitable for biomarker discovery studies.

## 2. Materials and Methods

### 2.1. Plasma Samples

**Figure 1:**
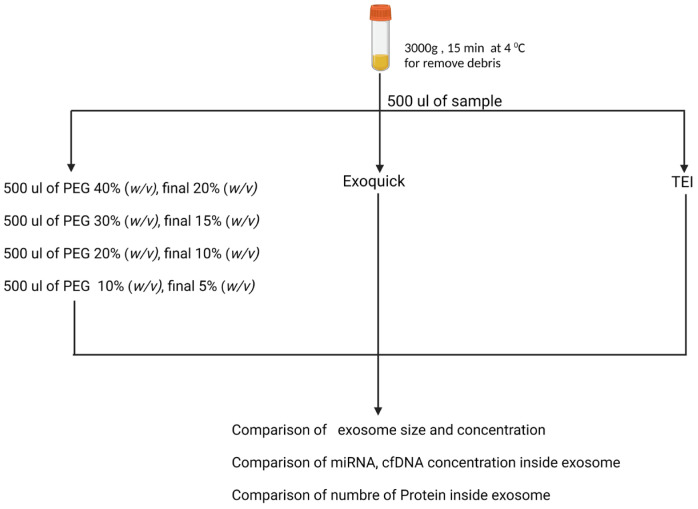
Experiment design of exosome isolation using different concentrations of PEG, ExoQuick precipitation, and Total Exosome Isolation (TEI).

Plasma specimens were collected from three female and three male healthy volunteers. The study was supported by the Ethics Committee of Chulalongkorn University Hospital. The blood samples were drawn into an EDTA tube. The samples were centrifuged at 1500 rpm for 10 min, and the subsequently collected plasma was pooled together for further exosome isolation. Plasma samples were centrifuged at 3000× *g* for 15 min. In the present study, exosomes were isolated using different methods, including precipitation by distinct concentrations of PEG 8000 (5, 10, 15, 20% (*w*/*v*)) (Sigma Aldrich, 3015, St. Louis, MO, USA), and with exosome isolation kits (Total Exosome Isolation TM (TEI) reagent from Life Technologies (Waltham, MA, USA) and ExoQuick from SystemBiosciences (Palo Alto, CA, USA). We characterized the size and concentration by NTA, and then determined their cfDNA, miRNA, and protein as shown in Figure 1.

### 2.2. Exosome Isolation

Prior to isolating exosomes, plasma samples were pre-treated with 4 μL of thrombin at room temperature for 5 min. The plasma samples were subsequently centrifuged at 10,000× *g* rpm for 5 min, followed by transferring a clear portion of plasma to a new tube. To eliminate DNA and RNA outside the exosome, the plasma samples were added 6.5 μL of 1500 unit of DNase I added for cfDNA isolation, and 0.5 mg/m/RNase A (Fisher Scientific) added for miRNA isolation, and were incubated at 37 °C for 15 min. PEG 8000 was prepared in 1 M NaCl to achieve 40, 30, 20, and 10% (*w*/*v*). Five-hundred microliters of pooled plasma was added to 500 μL of 40, 30, 20, and 10% (*w*/*v*) of PEG 8000 so that the final concentrations of PEG 8000 were 20, 15, 10, and 5% (*w*/*v*), respectively. Samples were centrifuged at 1500× *g* for 30 min followed by collecting a supernatant for further study. The exosome pellet was suspended in 250 μL of particle-free PBS and stored at −80 °C. Furthermore, isolation of exosomes by two commercial kits (ExoQuick^®^; SystemBiosciences (Palo Alto, CA, USA) and Total Exosome Isolation; Thermo Fisher Scientific (Waltham, MA, USA) was performed according to the manufacturers’ instructions.

### 2.3. Cell Free DNA Extraction

CfDNA was isolated by using the QIAamp^®^ Circulating Nucleic Acid Kit (Qiagen, Santa Clarita, CA, USA) according to the manufacturer’s instructions. Briefly, exosome supernatant (250 μL) was added to 800 μL of lysis buffer. After samples were heat inactivated at 56 °C in a water bath, 700 μL of ethanol (100%) was supplemented. Subsequently, the whole volume was sequentially centrifuged in one spin column. After the washing steps had been performed according to the manufacturer’s instructions, 30 μL of AVE buffer was pipetted directly onto the column membrane, and it was incubated at room temperature for 1 min and then centrifuged at 8000× *g* rpm for 1 min to elute cfDNA. The cfDNA fragment size and quantity were elucidated with an Agilent 2100 Bioanalyzer and the Agilent High Sensitivity DNA chip, according to the manufacturer’s instructions.

### 2.4. ALU-115 Quantification by Using Real-Time PCR (qPCR)

cfDNA concentration and integrity in plasma samples were examined by measuring ALU repeats (ALU-115 bp) using a ALU-115 primer set (Forward: CCTGAGGTCAGGAGTTCGAG and Reverse: CCCGAGTAGCTGGGATTACA) (Invitrogen, Life technologies, Waltham, MA, USA) The qPCR was performed using universal SYBR Green Master Mix (Invitrogen, Life technologies, Waltham, MA, USA) in a final volume of 25 μL comprising 12.5 μL of 1 × SYBR Green, 0.5 μL of 0.2 μM PCR forward and reverse primers, 5 μL DNA template, and 6.5 μL of RNase-free water. The thermal cycling condition was pre-activation of DNA polymerase at 95 °C for 15 min, followed by 40 cycles of denaturation at 94 °C for 15 s, annealing at 64 °C for 30 s, extension at 72 °C for 30 s, and then final extension at 72 °C for 10 min using StepOne™ real-time PCR (Applied Biosystems one step, Waltham, MA, USA).

### 2.5. miRNA Extraction and miR-122 and miR-16 Expression by Using Real Time PCR

The miRNA isolation was performed using the Serum/blood Kit (QIAGEN, Hilden, Germany) following the manufacturer’s instructions. Briefly, 250 μL of resuspended exosomes was processed with miRNA isolation columns and buffers provided by the manufacturer. A final volume of 30 μL miRNA solution was collected from each sample using the supplied elution buffer. The miRNA was reverse-transcribed using Taqman miRNA assays which were used to perform quantitative real time PCR (qPCR) (TaqMan^®^ Universal Master Mix II, no UNG, Applied Biosystems) for miR-122 (Assay ID: 002245) and miR-16 (Assay ID: 000391) according to the manufacturer’s instructions. In short, a total final volume of 15 μL reaction with 10 μL of total miRNA, RT mixture and primers was incubated at 16 °C for 30 min, 42 °C for 60 min, and 85 °C for 5 min. The miRNA expression levels were quantified with a 7500 ABI qPCR System (Applied Biosystems). 

### 2.6. Nanoparticle Tracking Analysis (NTA)

Five microliters of exosome suspensions was diluted with 1 mL of free-particle PBS and was examined using a Nanosight NS300 (NanoSight Ltd., Malvern Instrument). A video of 30 s duration was taken with a frame rate of 30 frames/s, and particle movement was analyzed using NTA 3.1 software.

### 2.7. Western Blot Analysis

Three microliters of exosome solution was denatured in 5 X sodium dodecyl sulfonate (SDS) buffer and subjected to Western blot analysis (12% SDS-polyacrylamide gel electrophoresis) using rabbit polyclonal antibody CD63 (mouse anti-human CD63, clone MX-49.129.5, Abcam, Cambridge, MA, USA), CD9 (mouse anti-human CD9, Abcam), and mouse monoclonal antibody TSG101 (clone 30A9, Abcam, Cambridge, MA, USA). The proteins were visualized on the Odssay system (RI technology, Rochester, NY, USA).

### 2.8. Sodium Dodecyl Sulfate Polyacrylamide Gel Electrophoresis (SDS-PAGE) and Proteomic Analysis Using LC-MS/MS

One hundred microliters of resuspended exosome was added to an equal amount of 2% SDS and sonicated at 40% amplitude for 1 min. Ten microliters of samples were separated by SDS-PAGE using 12.5% SDS gels and stained with a Coomassie Brilliant Blue G-250-based stain (Thermo Fisher, Waltham, MA, USA). Serial gel slices were excised, diced into smaller fragments, destained with 50% acetonitrile in 25 mM NH_4_HCO_3_, and dried. Samples were reduced with 10 mM dithiothreitol (DTT) (Merck, Kenilworth, NJ, USA) in 25 mM NH_4_HCO_3_ at 56 °C for 1 h and alkylated with 55 mM iodoacetamide (IAA) (GE Healthcare, Chicago, IL, USA) for 45 min at room temperature. 

In-gel trypsin digestion was performed using 12.5 ng/μL of sequential graded modified porcine trypsin (Promega) diluted in 25 mM NH_4_HCO_3_ at 37 °C overnight. Peptides were extracted with 0.5% formic acid and 50% acetonitrile. Following evaporation of acetonitrile, peptides were purified using a ZipTipC18 column (Millipore, Billerica, MA, USA). The volume of each eluted sample was reduced in a Speedvac to 5 μ of evaporated acetonitrile and adjusted to 20 μL with 0.1% formic acid prior to LC-MS/MS analysis. Peptides were separated by nano-liquid chromatography (EASY-nLC 1000, Thermo Fisher Scientific) coupled to a mass spectrometer (Q Exactive Plus Hybrid Quadrupole-Orbitrap, Thermo Fisher Scientific, Waltham, MA, USA) through an EASY-Spray nanoelectrospray ion source (Thermo Fisher Scientific, Waltham, MA, USA). The MS methods included a full MS scan at a resolution of 70,000 followed by 10 data dependent MS2 scans at a resolution of 17,500. The full MS scan range of 200–2000 *m*/*z* was selected, and precursor ions with the charge states of +1 or greater than +8 were excluded. Normalized collision energy of HCD fragmentation was set at 28%. Raw LC–MS/MS files were searched using Maxquant against Human UniProt proteins.

### 2.9. Statistical Analysis

Data from three independent replications were expressed as mean ± SD. One-way analysis of variance (ANOVA) followed by a Tukey’s analysis was used to investigate the differences between groups. *p* < 0.05 was considered to be statistically significant.

## 3. Results

### 3.1. Characterization of Exosomes from Different Concentrations of PEG Precipitation Conditions and Two Commercial Kits

**Figure 2:**
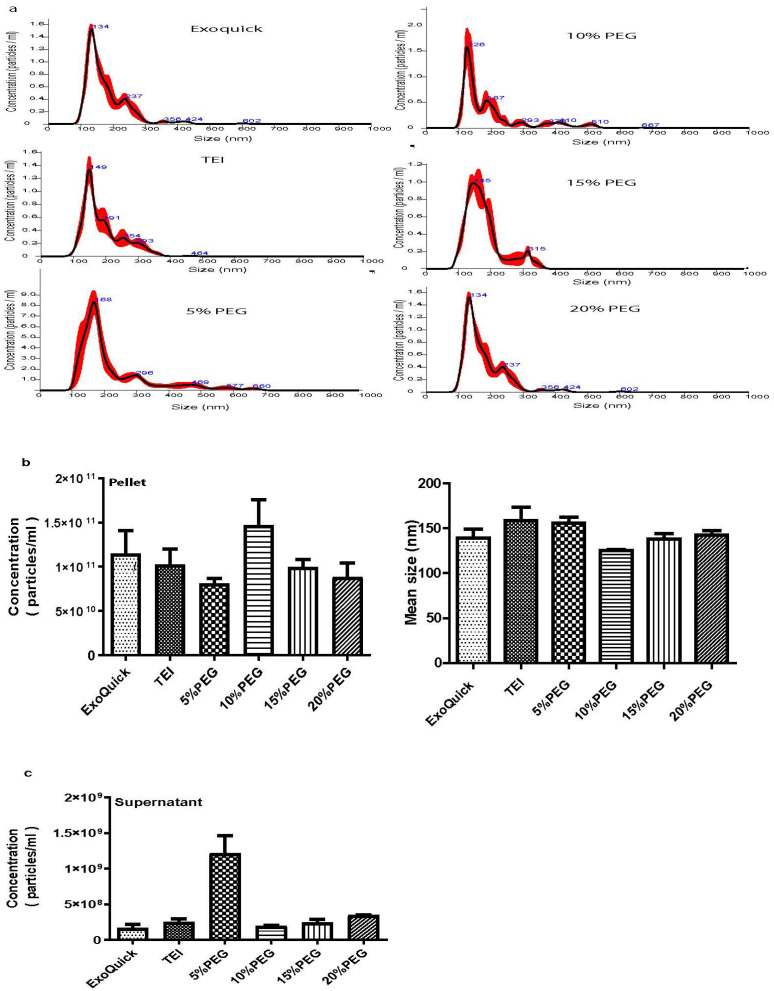
Characterization of exosomes purified by ExoQuick, TEI, and different concentrations of PEG. (**a**) Nanoparticle tracking analysis measurements. (**b**) Concentration of particles and mean size of exosome. (**c**) Concentration of particles in supernatant after precipitation. Data are presented as mean ± SEM of three independent experiments.

**Figure 3:**
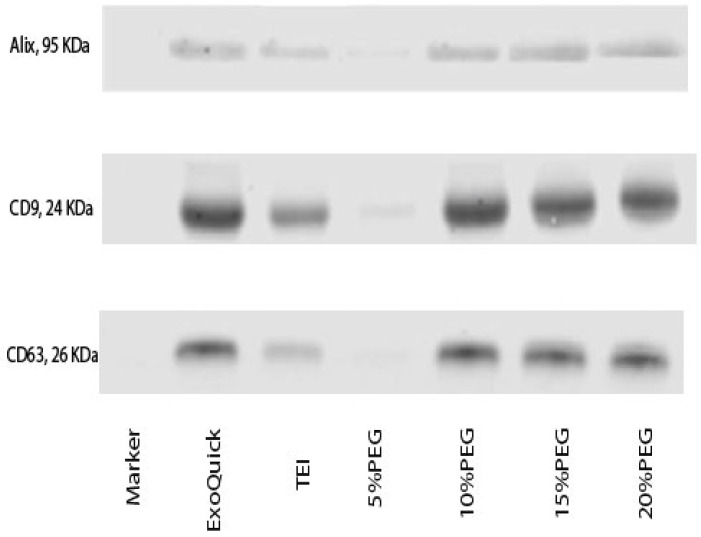
Western blot analyses with specific antibodies against 3 exosome markers: Alix, CD9, and CD63 from exosomes purified by using ExoQuick, TEI, and different concentrations of PEG.

To evaluate the efficiency of isolation using different concentrations of PEG and two commercially available kits (ExoQuick and TEI), we used pooled plasma samples for three experimental replicates and evaluated the size distribution and concentration of particles by using NTA. The size distribution using ExoQuick and TEI had the mean range of 134 and 149 nm in diameter, respectively, whereas the size distributions using PEG at concentrations of 5, 10, 15, and 20% (*w*/*v*) were 168, 126, 146, and 134 nm in diameter, respectively, as shown in Figure 2a. These results suggest that higher PEG concentrations can precipitate smaller extracellular vesicles. The concentration of particles demonstrated that the concentration of particles using 10% PEG was higher than with ExoQuick and TEI, as shown in Figure 2b. We noticed that the concentration of particles using PEG at 15 and 20% was lower than with 10% PEG, whereas the lowest concentration was seen with 5% PEG. In the supernatant after precipitation, the concentration of particles in 5% PEG was the highest, which corresponds to the aforementioned results, confirming that exosomes were not well-precipitated by 5% PEG. On the other hand, we found a small quantity of particles in the supernatants of isolation by ExoQuick, TEI, 10, 15, and 20% PEG, as depicted in Figure 2c. To verify exosomes, we determined the protein markers (CD63, CD9, and ALIX) of exosomes by Western blot analysis using an equal amount of sample after isolation. This result is consistent with the analysis from NT that 10% PEG and ExoQuick had a higher intensity of CD9, CD63, and ALIX than TEI and 5% PEG (Figure 3). These findings are consistent with David et al. 2016 [4] and Anna et al. 2018 [5] who reported that the particles of exosomes in tissue culture supernatant drop at higher concentrations of PEG. This result confirmed that 10% PEG can precipitate as efficiently as the two commercial kits.

### 3.2. Exosomal Cell Free DNA (cfDNA) and miRNA Analysis

**Figure 4:**
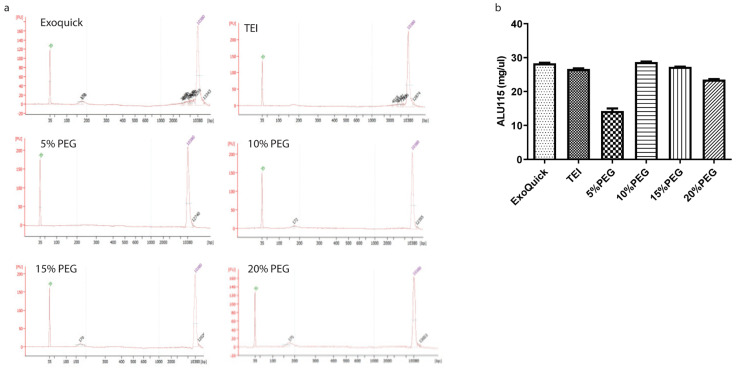
Bioanalyzer images of exosome isolated cfDNA from ExoQuick, TEI, and different concentrations of PEG. (**a**) The desired cfDNA fragment sizes peak is 170–176 bp; marked with an arrow. (**b**) Plasma ALU-115 repeats (ng/mL). Data are presented as mean ± SEM of three independent experiments.

**Figure 5:**
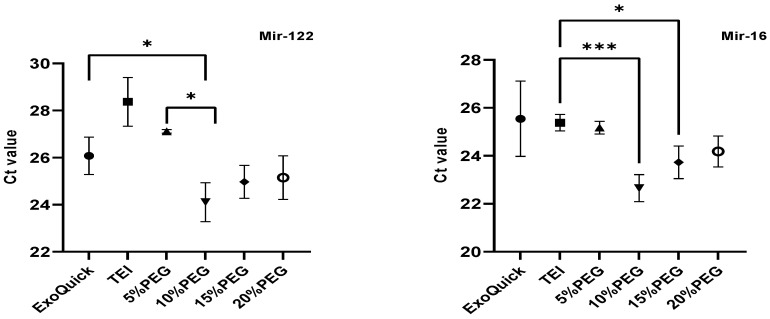
miR-122 and miR-16 Ct value expression. Data are presented as mean ± SEM of three independent experiments. (*); *p*-value ≤ 0.05, (***); *p*-value ≤ 0.001.

cfDNA is fragmentary DNA released into the blood. cfDNA can be detected in healthy individuals as well as in cancer patients, and it is also found in exosomes [6]. The differences in cfDNA fragment sizes can indicate biological activity. Short fragments (160–200 bp) are present from apoptotic cells and long fragments of cfDNA over 2–6 Kb are present from necrotic cells [7,8]. Moreover, cfDNA belong to DNA repeat sequences of arthrobacter luteus sequences (ALU), that is, short, interspersed elements [9]. Therefore, we determined fragment size of cfDNA by using bioanalyzer and amplicons of ALU115 as representative of total cfDNA concentration by qPCR. In Figure 4a, cfDNA fragments had sizes of 170 to 176 bps and were detected in ExoQuick, 10, 15, and 20% PEG. Interestingly, both TEI and 5% PEG were unable to detect the cfDNA size, perhaps due to the concentration of particles being low in cfDNA yield. The cfDNA fragment sizes isolated from ExoQuick, 10, 15, and 20% PEG were 176, 172, 170, and 175 bp, respectively. The levels of ALU115 of cfDNA in exosomes among the different methods were not significantly different, but interestingly 10% PEG had an equal level compared to ExoQuick, and it was slightly higher than TEI and 5% PEG (Figure 4b). We also measured ALU115 in supernatants after precipitation and found that the levels of ALU155 in TEI and 5% PEG were higher than ExoQuick and 10–20% PEG. MiRNAs found in exosomes were thought to be important effectors of intercellular communication [10]. Next, we evaluated miR-16 and miR-122 recoveries using qPCR. In the present study, we used equal volumes of miRNA from the different precipitation methods in order to compare the Ct value because there are no established miRNAs in blood to be used as endogenous housekeeping controls, and miR-16 in 10% PEG was significantly lower than using ExoQuick and TEI (Figure 5) [11]. The detected Ct value of both miR-122 and miR-16 in 10%PEG was significantly lower than exoquick and TEI. Whereas, TEI and 5% PEG had higher Ct values than other methods. These results indicate that miRNA abundance recovery from 10% PEG is higher than those using ExoQuick and TEI, suggesting that 10% PEG is more suitable for clinical samples and as a lower cost method for isolating miRNAs when compared to commercially available kits.

### 3.3. Proteomics Analysis 

**Figure 6:**
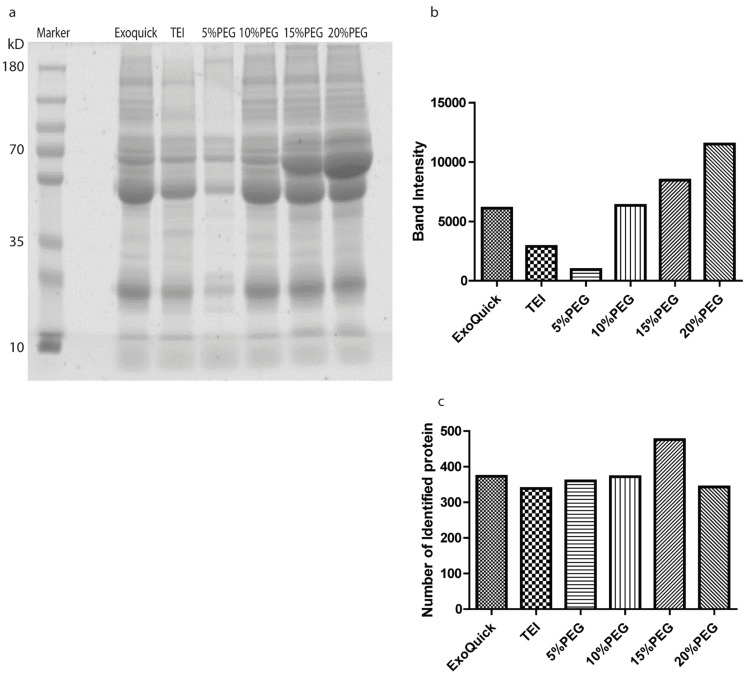
Proteomics results. SDS-PAGE of exosomes purified by ExoQuick, TEI, and different concentrations of PEG (**a**) and total band intensity (**b**). Number of identified proteins by using mass spectrometry (**c**).

**Figure 7:**
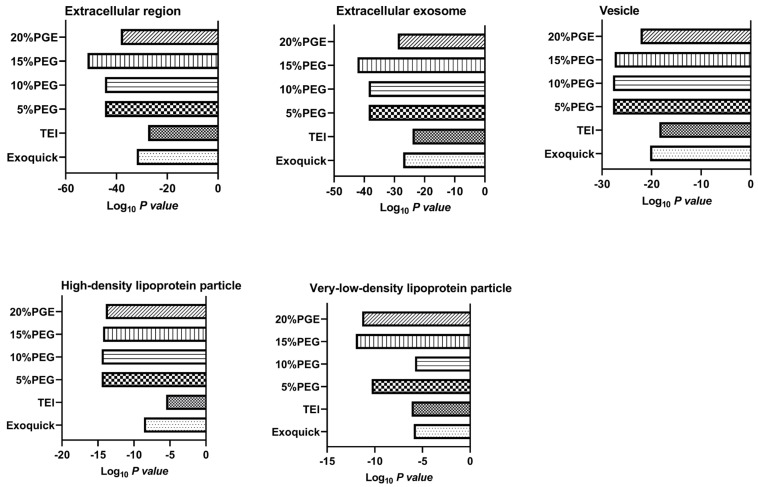
Gene oncology of exosomes purified by ExoQuick, TEI, and different concentrations of PEG.

The protein profiles for the different precipitate methods of isolation were analyzed using LC-MS/MS. Figure 6 shows protein bands for the different methods and measured intensity bands. We found that the band intensity of Coomassie blue in 20% PEG was the highest among the methods. We expected that increasing concentrations of PEG would tend to increase the abundance of proteins in plasma such as albumin and immunoglobin (molecular weight 50–70 kDa), as shown in SDS-PAGE gel. This result is similar to David et al. (2016) [4] for precipitation of off-target proteins in tissue cell culture with increasing concentrations of PEG (Figure 6a,b), whereas 10% PEG and ExoQuick were not different but TEI had a lower intensity band. After that, we cut each band into 24 gel pieces and did gel digestion. Peptides were submitted to LC/MSMS. We found number of identified proteins 372, 339, 360, 371, 475, and 341 from ExoQuick, TEI, 5, 10, 15, and 20% PEG, respectively (Figure 6c). We further to investigate the biological roles of cellular components were performed by using STRING PATHWAY (Figure 7). All identified proteins from different concentrations of PEG were involved in extracellular exosomes, extracellular regions, and vesicles, whereas we found the number of identified proteins involved in low-density lipoprotein particles and high-density lipoproteins in different concentrations of PEG was more than with ExoQuick and TEI. The data from our study suggest that the contaminant of lipoproteins particles, albumin and other abundant proteins found in the precipitate obtained from PEG precipitation could affect the shotgun proteomic approaches for detecting low-abundance proteins. These data suggested that PEG solutions can enrich exosomes and also can precipitate proteins in plasma effectively.

## 4. Discussion

Exosomes serve as a liquid biopsy tool for clinical research because they can protect internal material contents from degradation by DNase, RNase, and protease enzymes. In this study, we compared different concentrations of PEG 8000/1M NaCl with two commercial kits (ExoQuick and TEI) for plasma exosome isolation combined with downstream applications. Our main aim was to optimize the concentration of PEG for suitable downstream clinical and translational applications.

The isolation of exosomes from pooled human serum samples using different concentrations of PEG 8000 (5, 10, 15, and 20%) suggested that 10% PEG solution can isolate exosomes as effectively as ExoQuick and it shows better performance than TEI. 

Our results seem in contrast to a study conducted by Mark et al. 2020 which reported that TEI exosome recovery had greater yield than 8% PEG 6000.

However, our results showed that the concentration of PEG 8000 appears to play a key role in determining the quality and quantity of exosomes. Our finding is in concordance with a previous study about PEG-based precipitation of exosomes from cell culture supernatants achieved through a final 10% PEG solution. The study found that 10% PEG 10,000 was more efficient in exosome recovery compared to 5% and 7% PEG [12]. The verification was carried out by Western blot analysis targeting exosome marker proteins (CD63, CD9, and ALIX). We found the band intensity of 10–20% PEG was equal to ExoQuick, while it had a higher band intensity than TEI and 5% PEG. Furthermore, optimizing the percentage of PEG solution was effective for the recovery of exosomes because higher percentage PEG solutions have phenomenon to exosome aggregation coated with silk-like PEG films and other extracellular vesicles that was observed by transmission electron microscope effect to exosome recovery and purity [13]. In this work, we further examined and compared cfDNA and miRNA recovery by the different methods for translational downstream applications. Surprisingly, cfDNA fragment size was not detected by using bioanalyzer in TEI and 5% PEG due to low yield. However, the detection of cfDNA using ALU115 by qPCR was able to quantify total cfDNA. In the liquid biopsy era, total cfDNA is being used for markers of cancer and stage of disease. In our results, we found that totals of cfDNA in 10% PEG and ExoQuick are not different and had lower amounts than in 15–20% PEG. This result correlates with the concentration of particles and is in agreement with the report conducted by Garicia-Romero, N et al. 2019 [14]. They observed similar gDNA concentrations as with PEG and ExoQuick. For miRNA analysis, TEI and 5% PEG had high Ct values in detection of miR-122 and miR-16 which indicated the concentration of miRNA presented in exosomes was low. Interestingly, Ct values of miR-122 and miR-16 expression for 10–20% PEG were lower than ExoQuick and TEI, which suggested that totals of miRNA in 10–20% PEG were higher than with the two commercial kits. Although the concentrations of particles in 10% PEG and the two commercial kits are quite different, there was no significant effect on miRNA levels inside exosomes. However, in relation to free miRNA and miRNA bound to Argonaute-2 in plasma [15], we treated samples with RNase A before extracting miRNA. Hence, it is possible that there was no contamination of free miRNA. Our data strongly suggest that exosome samples obtained with different precipitation methods are almost devoid of contamination with other circulating miRNAs.

For proteomics, we loaded the same volume for the different precipitation methods. Our data show that 20% PEG has the highest band intensity because PEG can precipitate immunoglobulins or abundant proteins in plasma [5], so increasing concentrations of PEG can precipitate more immunoglobulin and other proteins in serum. In addition, GO function enrichment analysis of cellular components suggested that the identified proteins from PEG solutions were mainly involved in extracullular exosomes, extracullular regions, and vesicles, more than the two commercial kits, but were highly contaminated with other proteins in serum such as lipoprotein, immunoglobulin, and so on.

In conclusion, this study provides evidence that 10% PEG 8000 could be a method of choice for exosome isolation and an alternative to the commercial kits. PEG solution is also useful for exosomal RNA and cfDNA analysis, while being unsuitable for protein analysis due to lipoprotein contamination and protein aggregation. Overall, PEG provides a simple and inexpensive means for harvesting exosomes from biological fluids and clinical samples, and it can be used easily in any laboratory.

## Data Availability

The data presented in this study are available on request from the corresponding author.
